# Efficacy and safety of femtosecond laser-assisted cataract surgery versus conventional phacoemulsification for cataract: a meta-analysis of randomized controlled trials

**DOI:** 10.1038/srep13123

**Published:** 2015-08-13

**Authors:** Xiaoyun Chen, Wei Xiao, Shaobi Ye, Weirong Chen, Yizhi Liu

**Affiliations:** 1 State Key Laboratory of Ophthalmology, Zhongshan Ophthalmic Center, Sun Yat-sen University, Guangzhou 510060, People’s Republic of China

## Abstract

The aim of this study was to evaluate the efficacy and safety of femtosecond laser-assisted cataract surgery (FLACS) versus conventional phacoemulsification cataract surgery (CPCS) in the treatment of cataract. Randomized controlled trials (RCTs) were searched in PubMed, Embase and the Cochrane Central Register of Controlled Trials. Nine qualified studies with a total of 989 eyes were included. Compared with CPCS, FLACS significantly reduced mean phaco energy and effective phacoemulsification time (EPT) required in the surgery. Central corneal thickness (CCT) was significantly lower in FLACS at 1 day of follow-up, but CCT and corneal endothelial cells count was comparable at 1 week of follow-up or longer. FLACS achieved a better visual outcome at postoperative 1 week and 6 months, but the difference was not significant at postoperative 1–3 months. Regard to surgical complications, the incidences of intraoperative anterior capsule tear, postoperative macular edema and elevated intraocular pressure were similar. In conclusion, femtosecond laser pretreatment can reduce phaco energy and EPT, which may reduce the heat damage to ocular tissues by ultrasound. This novel technique might be beneficial for patients with dense cataract and/or low preoperative endothelial cell values. Well-designed RCTs with longer follow-up are still necessary to provide more reliable evidence.

Cataract surgery is one of the most commonly performed surgery in the world and the number of individuals with cataracts is predicted to reach 30 million by the year 2020^1^. This number will continue to grow as the population ages. With the improvement of living standards, more and more patients pursue surgery at the early stage of cataract in order not to endure visual impairment. Therefore, cataract surgeons are facing increasingly high patient expectations for visual acuity outcome after surgery. Nowadays, the goal of cataract surgery is to achieve near emmetropia.

Phacoemulsification is the standard surgery procedure for cataract in the developed countries. It introduces ultrasound to fragment and emulsify the cataract during surgery. The incision is smaller than extracapsular cataract extraction (ECCE), which allows rapid visual rehabilitation, low surgery-induced astigmatism, and complication reduction^2^. However, ultrasound meanwhile creates heat and damage to corneal endothelium and results in cornea edema. In the last several years, advances in phacoemulsification technique and technology, such as intraocular lens (IOL) technology, energy delivery, system fluidics and instrumentation, have made cataract surgery more and more safe and efficient^3^,^4^,^5^. Despite these advances, sight-threatening complications, including postoperative corneal edema^6^, posterior capsular rupture^7^, cystoid macular edema^8^,^9^ and endophthalmitis^9^,^10^ still occur.

In recent years, femtosecond laser (FSL) has been introduced into phacoemulsification cataract surgery to perform corneal incisions, capsulorhexis, and nuclear fragmentation^11^. The application of FSL in cataract surgery may be one of the biggest revolutions in the field of cataract in the latest several years. Some researchers proposed FSL has the potential to offer significant advantages over conventional phacoemulsification (CP) and will be the standard method of cataract surgery in ten years^12^. The major advantage of FSL-assisted cataract surgery (FLACS) is the reduction of phacoemulsification energy required in the surgery. Numerous clinical studies have reported that using FSL to perform nuclear fragmentation before phacoemulsification significantly reduces the amount of ultrasound energy and effective phacoemulsification time (EPT) required in the surgery^13^,^14^,^15^,^16^,^17^. It is well known that the application of ultrasound during phacoemulsification can lead to corneal endothelial cell damage due to mechanical trauma from sonic waves and thermal injury. Therefore, several studies have reported that the application of FSL can alleviate corneal endothelial cell loss and corneal edema in the early postoperative period^13^,^18^,^19^,^20^. Another advantage of FLACS is able to create a more circular and precise capsulorrhexis, which can facilitate phacoemulsification and IOL implantation, and offer more accurate refractive outcomes after surgery^21^,^22^,^23^,^24^. However, some researches also suggested FLACS has not provide advantage over conventional CP, and it will not be a standard method for cataract surgery ten years from now^25^. Several studies have verified that FLACS does not achieve a better postoperative visual outcome than conventional phacoemulsification cataract surgery (CPCS)^19^,^26^,^27^. Some studies also found comparable results in corneal endothelial cell loss and corneal edema compared with CPCS after long-term follow-up^20^,^28^. Moreover, a body of studies even reported that clinically relevant complications are more common in the FLACS cases, such as anterior capsule tear and anterior capsule adhesions or tags^29^,^30^.

Is FLACS safer and more effective than CPCS ? The inconsistent results could not reach a conclusion for clinical practice. There are still many unanswered questions. Small-scale population and short-term follow-up limit the ability to adequately assess the efficacy and safety factors of FLACS. To our best knowledge, the data about FLACS have not yet been systematically evaluated and reported. Therefore, we performed a meta-analysis of all available randomized controlled trials (RCTs) to compare the efficacy and safety of FLACS with CPCS.

## Results

### Literature search

**Figure 1:**
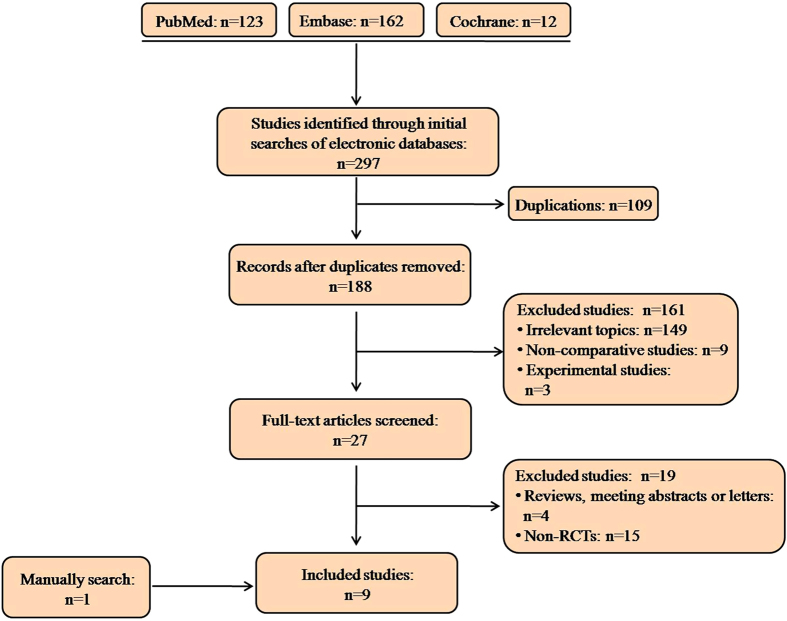
Flow chart of literature search and study selection.

Our search and selection process is summarized in Fig. 1. A total of 297 articles were initially identified. After removing duplications, the abstracts of the remaining studies were reviewed and 27 articles with potentially relevant trials were further identified in their full texts. Eight randomized controlled trials (RCTs) were eligible after full text screen. We also identified 1 qualified study though manually checking the reference lists of the retrieved studies. Therefore, 9 RCTs published from 2011–2014 were finally included in this meta-analysis.

### Characteristics of the included studies

We included 9 RCTs^13^,^17^,^18^,^19^,^23^,^26^,^31^,^32^,^33^ in this review. All studies were published in English, since 2011. Three studies were conducted in Germany, three in Hungary, two in Italy, and one in India. Table 1 summarizes the characteristics of included studies. In total, 989 eyes were included, 512 in the FLACS group and 477 eyes in the CPCS group. The average age of participants ranged from 58.5–71.3 years. Sample sizes ranged from 20 to 104, and the follow-up time ranged from 1 day to 12 months.

### Risk of bias in included studies

Results of the risk of bias assessments of included studies were presented in Fig. S1 in the supplement. For selection bias, 5 studies used a computer-generated randomization scheme^18^,^23^,^26^,^32^,^33^, other 4 studies did not state the randomization method explicitly. For performance and detection biases, all studies were judged to be at high risk of bias in Blinding of participants and personnel domain, because the surgical procedures were obviously different. Only two studies were double-masked (participants and outcome assessors)^18^,^33^, and unclear in the remaining studies. For attrition bias, the patients in all studies except one^18^ achieved 90% power, and the numbers of participants who exited the study were reported clearly and were unlikely to affect the outcome. Therefore, we judged the attrition bias to be of low risk in all studies. We could not comment on the risk of selective reporting because we did not have access to the study protocols, but all outcomes described in respective methodologies were reported.

### Efficacy analysis

#### Postoperative visual acuity

**Figure 2:**
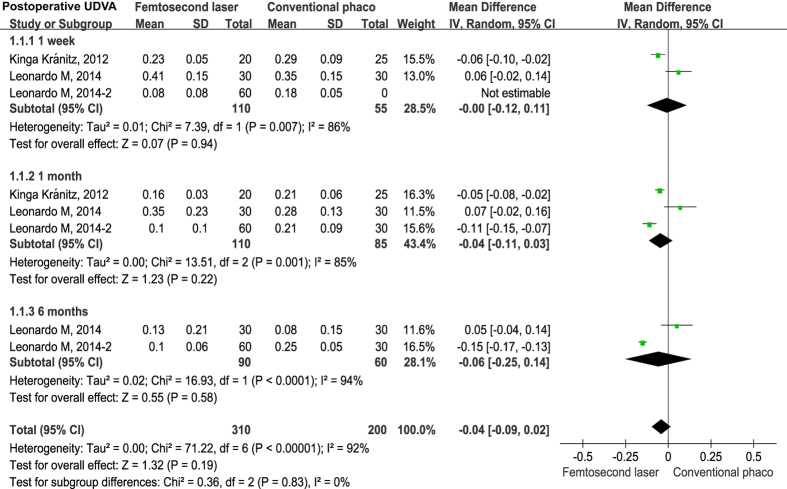
Forest plots displaying the effect of FLACS *versus* CPCS on uncorrected distance visual acuity (UDVA) at postoperative 1 week, 1 month and 6 months.

**Figure 3:**
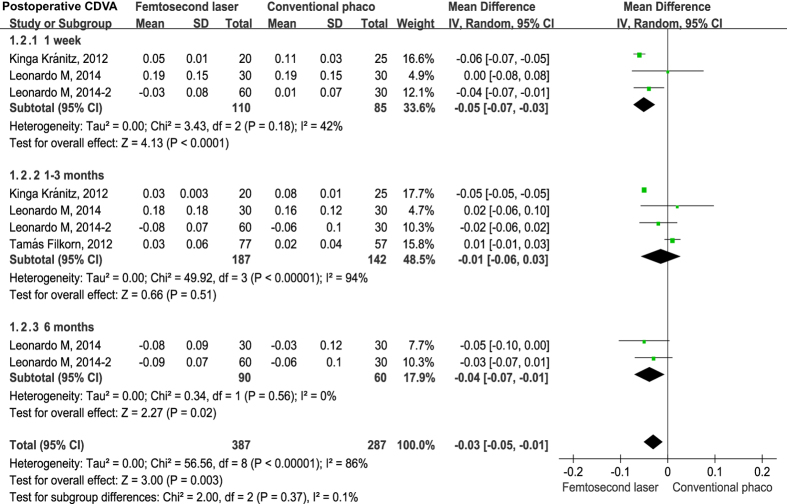
Forest plot exhibiting the effect of FLACS *versu*s CPCS on corrected distance visual acuity (CDVA) at postoperative 1 week, 1–3 months and 6 months.

Seven studies reported postoperative uncorrected distance visual acuity (UDVA)^19^,^23^,^33^ and corrected distance visual acuity (CDVA)^19^,^23^,^26^,^33^ as a logarithm of the minimal angle of resolution (logMAR) at different time points. Examination of the forest plots revealed no statistically significant differences in postoperative UDVA between FLACS *versus* CPCS groups during the follow-up period (Fig. 2). However, the differences of postoperative CDVA were statistically significant at 1 week (mean difference = −0.05, 95% CI −0.07 to −0.03, *P* < 0.001) and 6 months (mean difference = −0.04, 95% CI −0.07 to −0.01, *P* = 0.020), but the differences were not significant at 1–3 months (mean difference = −0.01, 95% CI −0.06 to 0.03, *P* = −.051) (Fig. 3).

#### Postoperative corneal endothelial cell counts

**Figure 4:**
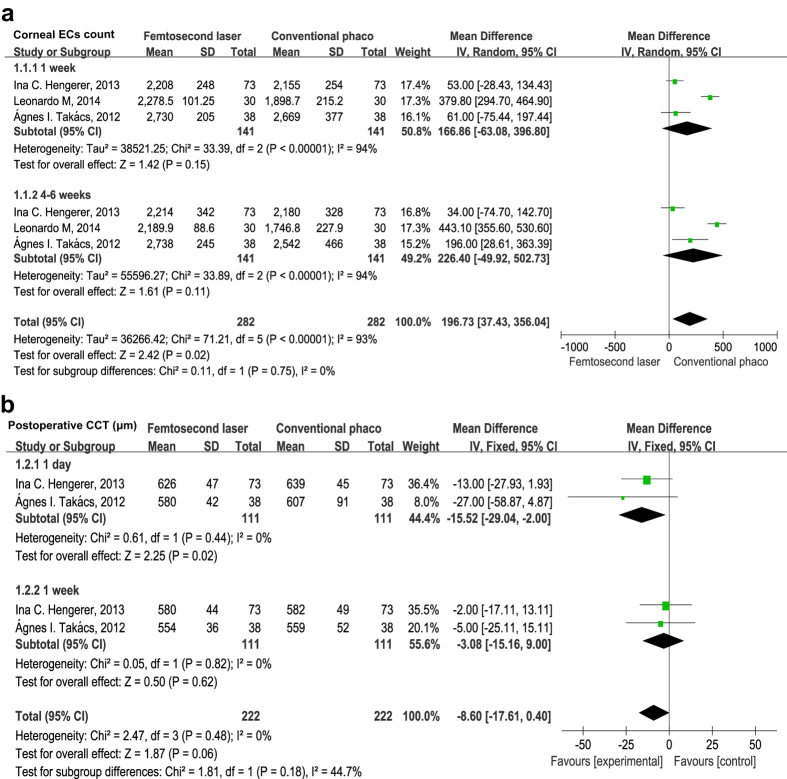
(**a**) Forest plots showing the effect of FLACS *versus* CPCS on the corneal endothelial cell counts at postoperative 1 week and 4–6 weeks. (**b**) Forest plots displaying the effect of FLACS *versus* CPCS on the central corneal thickness (CCT) at postoperative 1 day and 1 week.

For comparison of postoperative corneal endothelial cell counts between FLACS *versus* CPCS groups, data were collected from 3 studies^13^,^18^,^19^. The pooled data showed that corneal endothelial cell counts were slightly higher in the FLACS group than CPCS group (mean difference = 196.7, 95% CI 37.43 to 356.0, *P* = 0.020) (Fig. 4a). However, when corneal endothelial cell counts were further divided into at postoperative 1 week and 4–6 weeks two different time points, the differences were not statistically significant (mean difference = 166.9, 95% CI −63.08 to 396.8, *P* = 0.150; mean difference = 226.4, 95% CI −49.92 to 502.7, *P* = 0.110) (Fig. 4a).

#### Postoperative central corneal thickness (CCT)

Two studies^13^,^18^ including 444 eyes assessed postoperative CCT at two different time points, ranging from the first postoperative day to 1 week. The pooled data showed postoperative CCT was significantly lower in the FLACS group at postoperative day 1 (mean difference = −15.52 μm, 95% CI −29.04 to −2.00, *P* = 0.020) (Fig. 4b). However, the differences were not statistically significant at 1 week (mean difference = −3.08 μm, 95% CI −15.16 to 9.00, *P* = 0.620). These results suggest that FLACS causes less damage to the cornea and less corneal swelling than CPCS in the early postoperative period, but the differences are not significant after long-term follow-up.

#### Mean phaco energy, mean phaco time and effective phaco time (EPT)

**Figure 5:**
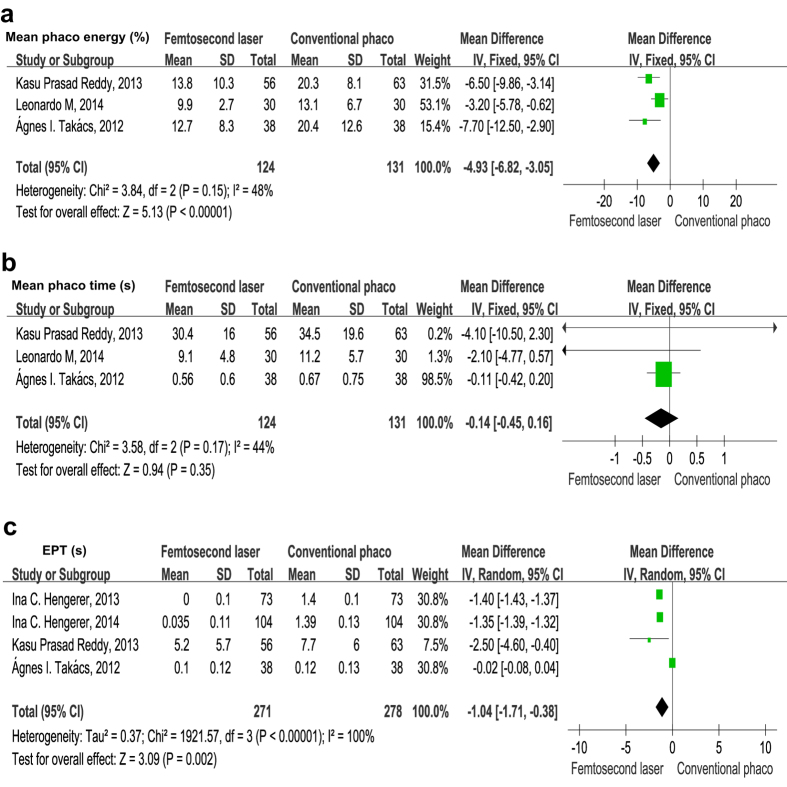
Forest plots revealing the effect of FLACS *versus* CPCS on mean phaco energy (**a**), mean phaco time (**b**), and effective phaco time (EPT) used in the surgery.

Three RCTs^17^,^18^,^19^ including 255 patients reported mean phaco energy and mean phaco time. Examination of the forest plots revealed mean phaco energy was much higher in the CPCS group than in the FLACS group, the differences were statistically significant (mean difference = −4.93%, 95% CI −6.82 to −3.05, *P* < 0.001) (Fig. 5a). Nevertheless, the forest plots demonstrated that there were no statistically significant differences in mean phaco time between two group (mean difference = −0.14 s, 95% CI −0.45 to 0.16, *P* = 0.350) (Fig. 5b).

Pooling the data from 4 studies^13^,^17^,^18^,^31^ that assessed EPT in 549 eyes all showed there was a statistically significant reduction in the EPT in the FLACS group compared with the CPCS group (mean difference = −1.04 s, 95% CI −1.71 to −0.38, *P* = 0.002) (Fig. 5c). These results indicate that the use of FSL in cataract surgery leads to a much lower EPT compared to the standard procedure.

#### Capsulorhexis quality measures

**Figure 6:**
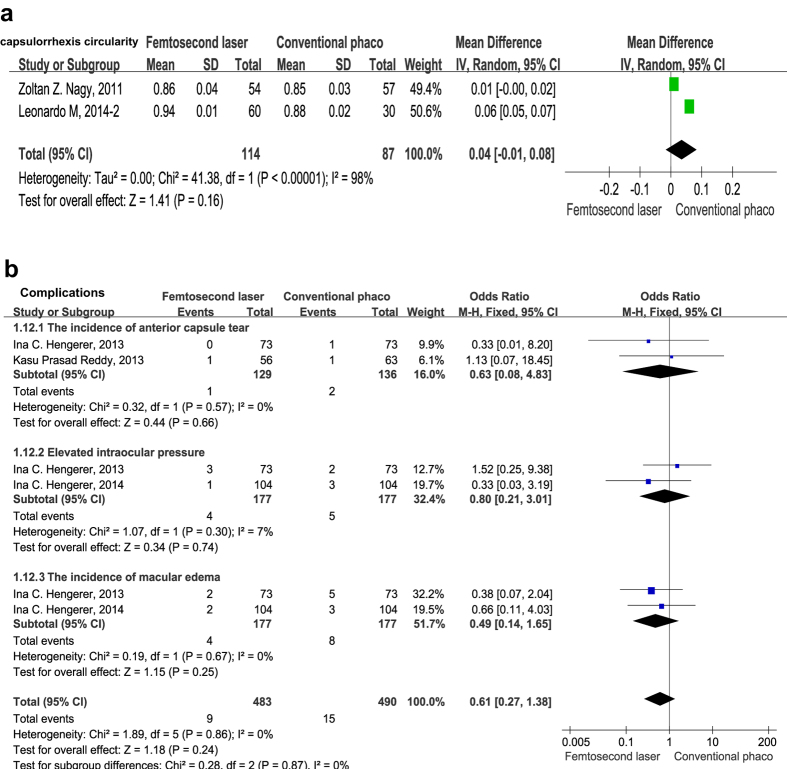
(**a**) Forest plots representing the effect of FLACS *versus* CPCS on the circularity of capsulorrhexis at postoperative 1 week. (**b**) Forest plots showing the incidences of intraoperative and postoperative complications in FLACS *versus* CPCS.

Two studies^32^,^33^ evaluated the capsulorrhexis circularity which is a parameter used to determine the regularity of the shape of the capsulotomy. Value of 1.0 indicates a perfect circle. Analysis of these data demonstrated that circularity values were no statistically significant differences at 1 week between two groups (mean difference = 0.04, 95% CI −0.01 to 0.08, *P* = 0.160) (Fig. 6a).

### Safety analysis

Adverse events comparing FLACS and CPCS groups are showed in Fig. 6b. Anterior capsule tear is one of the most commonly reported intraoperative adverse events in FLACS. Previous studies have reported FLACS was associated with a significantly higher frequency of anterior capsule tear and anterior capsule adhesions or tag^29^,^30^. However, the pooled data of our analysis suggested FLACS did not show a higher rate of anterior capsule tear (OR = 0.63, 95% CI 0.08 to 4.83, *P* = 0.660) (Fig. 6b). The incidences of macular edema and elevated intraocular pressure (IOP) after uneventful cataract surgery are safety issues for this frequently performed operation. Therefore, we also compared the rate of macular edema and elevated intraocular pressure between two groups. The pooled data displayed that no significant differences were found in the incidence of macular edema and elevated IOP, with the pooled ORs being 0.49 (95% CI 0.14 to 1.65, *P* = 0.250), 0.80 (95% CI 0.21 to 3.01, *P* = 0.740) (Fig. 6b), respectively. These data imply that FLACS exhibits no effect on the occurrence of anterior capsule tear, macular edema and IOP when compared with CPCS.

## Discussions

FSL is a new and developing technology for cataract surgery. However, the efficacy and safety of FLACS have not yet been systematically evaluated. In this meta-analysis, the pooled results demonstrated that FLACS is superior to CPCS in the reduction of mean phaco energy and EPT. It achieves a better visual outcome at 6 months of follow-up. However, the number of corneal endothelial cells and the thickness of central cornea were comparable after 1 week. Additionally, FLACS does not gain a more precise capsulotomy *versus* CPCS. Regard to clinically relevant complications, the present study found that FLACS do not increase the incidences of intraoperative anterior capsule tear, postoperative macular edema and elevated intraocular pressure.

The functions of FSL in cataract surgery include circular capsulotomy formation, lens fragmentation, lens softening, and corneal incision creation. Lens fragmentation and softening with FSL before phacoemulsification is a primary advantage of FLACS over conventional surgery, especially for dense cataract. In this meta-analysis, FSL pretreatment leads to a significant reduction in phaco energy and EPT required during cataract surgery. This is consistent with all previous studies^13^,^17^,^18^,^19^,^29^,^31^. Phaco energy and time is the most significant risk factor for corneal endothelial cells and surrounding ocular tissues damage. Reducing phaco energy and time may minimize biomechanical damage to the cornea endothelial cells and result in an improvement in postoperative corneal edema. Since corneal edema is one of the most common early postoperative complications after phacoemulsification and may delay rapid visual rehabilitation. Therefore, we also evaluated the effect of FSL pretreatment on postoperative corneal edema and endothelial cell loss. However, although FSL pretreatment resulted in a significant reduction in corneal edema at postoperative day 1compared with CPCS, the differences were not significant in endothelial cell loss and CCT at 1week of follow-up or longer. Thus, from the current evidence, FLACS is only superior to CPCS in postoperative corneal edema in the early postoperative period. Given that FSL pretreatment can dramatically reduce phaco energy and EPT required in the surgery, FSL might be beneficial for patients with dense cataract or low preoperative endothelial cell values, such as Fuchs’ endothelial dystrophy, pseudoexfoliation, or history of trauma may particularly^34^. Certainly, large sample size studies with longer follow-up period are necessary to provide more reliable evidence.

The capsulotomy is known to be one of the most critical steps of phacoemulsification cataract surgery. An inappropriately constructed capsulorhexis may cause capsular tears, posterior capsular rupture, vitreous loss, as well as IOL tilt, rotation, decentration, and posterior capsular opacification development^24^,^35^,^36^. Therefore, we also systematically compared the quality of capsulorhexis. However, the data exhibited laser-cut anterior capsulotomy does not provide a more accurate capsulorhexis compared with manual cut capsules. Better refractive outcomes are most concerned by ophthalmologists and patients. Nowadays, the goal of cataract surgery is to achieve near emmetropia. In present study, we found FLACS achieves a better postoperative CDVA than CPCS at 1 week and 6 months, but UDVA was not different during the follow-up period. But only two studies reported UDVA and CDVA at 6 months^19^,^33^, so large sample size and well-designed studies are still necessary to provide sufficient evidence.

Anterior capsule tears is the most common intraoperative complication for FLACS^29^,^37^. The integrity of laser-cut anterior capsulotomy seems to be compromised by postage-stamp perforations and additional aberrant pulses, possibly because of fixational eye movements^29^. A learning curve may also account for the increased anterior capsule tear rate with FLACS^37^. However, our meta-analysis demonstrated that the rate of anterior capsule tear was not more frequent in the FLACS group. The surgeons become increasingly experienced with femtosecond laser may contribute to the low rate of anterior capsule tear. The occurrence of subclinical macular edema and elevated intraocular pressure after cataract surgery also have become safety issues for this operation^38^,^39^. Our pooled results showed FLACS does not increase the incidence of these clinically relevant complications. These data indicate that FLACS is considered a relatively safe technology for cataract surgery.

Although the results of our meta-analysis are credible and helpful for clinical decision-making for cataract management, it still has some limitations that should be taken into account. First, the heterogeneity of follow-up period and outcome definition made it impossible to extract the data from all the included studies. Second, this meta-analysis was restricted to data from published studies, so information bias could not be fully ruled out if studies with small sample-size or unpublished data exist. Thus, well-designed RCTs with longer follow-up period are still necessary to provide more reliable evidence.

In conclusion, our meta-analysis supports that FLACS is superior to CPCS in terms of mean phaco energy and EPT reductions, which might be beneficial for patients with dense cataract or low preoperative endothelial cell values. Corneal endothelial cell loss, corneal edema reduction, and the quality of capsulorhexis are similar to CPCS. The long-term visual outcome still needs large sample-size, well-designed RCTs to provide sufficient evidence. In addition, FLACS is a relatively safe technology for cataract surgery. Nevertheless, the cost and space of the femtosecond machine is another big limitation for the universal application of FSL in cataract surgery. Therefore, FLACS may not be the standard method for cataract surgery in the coming years. However, large patient populations, well-designed RCTs with longer follow-up period are necessary to update the findings of this analysis.

## Materials and Methods

### Literature-search strategy

We systematically searched PubMed, Embase, and the Cochrane Central Register of Controlled Trials (CENTRAL) from inception until June 2015. Our combined search terms were (femtosecond OR bladeless) AND (phaco OR phacoemulsification OR phakoemulsification) AND cataract. The details of search strategies were described in Appendix 1 (Supplementary information). Firstly, we used Endnote software to exclude the duplications. After that, we assessed titles and abstracts to remove apparently irrelevant studies. Finally, we retrieved full texts and assessed for eligibility. We also manually checked the reference lists of all retrieved studies and review articles to identify studies not found by the electronic searches.

### Inclusion and exclusion criteria

The selection of eligible studies was done by two authors (W. X and X. C) independently. Inclusion criteria were RCTs comparing the efficacy and safety of FLACS versu*s* CPCS in cataract patients who were at least 18 years old and elected to have routine cataract surgery. Abstracts from conferences, full texts without raw data, duplicate publications, letters to editors, and reviews were excluded.

### Data extraction and out-comes of interest

Two authors (W. X and X. C) extracted data and compared the results for differences. We resolved discrepancies by discussion. For efficacy analysis, the primary outcomes were UDVA and CDVA since both of them were most concerned by ophthalmologists and patients. The secondary outcomes included: 1) postoperative corneal endothelial cell counts and CCT: indicators of postoperative corneal edema which may delay rapid visual rehabilitation after cataract surgery; 2) EPT: a metric of the length of phacoemulsification time at 100% power in continuous mode; 3) mean phaco energy and mean phaco time, which are defined as the most significant risk factors for corneal endothelial cells and surrounding ocular tissues damage; and 4) the circularity of capsulorrhexis, which is a parameter used to determine the regularity of the shape of the capsulotomy with a value of 1.0 indicating a perfect circle. Additionally, safety was evaluated by considering the frequency of intraoperative and postoperative ocular complications, including anterior capsule tear, macular edema and elevated intraocular pressure. In accordance with Saldanha *et al.*^40^, each of the aforementioned outcomes was defined from the following five aspects in Appendix 2 (Supplementary information): 1) outcome name; 2) the specific measurement; 3) the specific metrics; 4) the method of aggregation; and 5) the time-point.

### Assessment of risk of bias

The risk of bias of included studies was assessed using the Review Manager 5.2 (Cochrane Collaboration, Oxford, UK). The assessment consists of six factors: random sequence generation and allocation concealment (selection bias), blinding of participants and personnel (performance bias), blinding of outcome assessment (detection bias), incomplete outcome data (attrition bias), and selective reporting (reporting bias). Each domain was graded as low, unclear, and high risk of bias according to the criteria outlined in the Cochrane Handbook for Systematic Review of Interventions. Two reviewers (W. X and X. C) independently assessed the risk of bias and resolved discrepancies by discussion.

### Statistical analysis

All statistical analyses were conducted using Review Manager 5.2. The weighted mean difference (WMD) and odds ratio (OR) were used to compare continuous and dichotomous variables, respectively. All the results were presented with 95% confidence intervals (CIs). For studies that presented continuous data as means and range values, the standard deviations were calculated using the method described by Hozo *et al.*^41^. Statistical heterogeneity among studies was explored using I^2^ tests. I^2^ values of 50% or more were considered substantial heterogeneity. The random-effects model was used if there was heterogeneity among studies; otherwise, the fixed-effects model was used.

## Additional Information

**How to cite this article**: Chen, X. *et al.* Efficacy and safety of femtosecond laser-assisted cataract surgery versus conventional phacoemulsification for cataract: a meta-analysis of randomized controlled trials. *Sci. Rep.* **5**, 13123; doi: 10.1038/srep13123 (2015).

## Supplementary Material

Supplementary Information

## Figures and Tables

**Table 1:** Characteristics of included studies.

First Author (year)	Center	Location	Groups	No. eyes	Sex (M/F)	Age (year)	FU (weeks)
Zoltán Z. Nagy *et al.* (2011)	single	Hungary	FLACS	54	15/39	65.0 ± 13.0	1
			CPCS	57	17/40	68.0 ± 15.0	
Ágnes I. Takács *et al.* (2012)	single	Hungary	FLACS	38	10/28	65.8 ± 12.4	4
			CPCS	38	15/23	66.9 ± 11.0	
Kinga Kránitz *et al.* (2012)	single	Hungary	FLACS	20	5/15	68.2 ± 10.8	48
			CPCS	25	2/23	63.6 ± 13.7	
Tamás Filkorn *et al.* (2012)	single	Germany	FLACS	77	NA	65.2 ± 12.6	9.72 ± 2.82 9.67 ± 2.66
			CPCS	57	NA	64.4 ± 12.4	
Ina C. Hengerer *et al.* (2013)	single	Germany	FLACS	73	27/46	70.9	12
			CPCS	73	27/46	70.9	
Kasu P. Reddy *et al.* (2013)	single	India	FLACS	56	30/26	58.5 ± 11.6	1 day
			CPCS	63	37/26	61.3 ± 9.7	
Ina C. Hengerer *et al.* (2014)	single	Germany	FLACS	104	46/58	71.3	24
			CPCS	104	46/58	71.3	
Leonardo M *et al.* (2014)	single	Italy	FLACS	30	NA	70.2 ± 2.9	24
			CPCS	30	NA	70.5 ± 3.2	
Leonardo M *et al.* (2014)-2	single	Italy	FLACS	60	NA	69.3 ± 3.2	24
			CPCS	30	NA	69.1 ± 3.9	

FLACS = femtosecond laser-assisted cataract surgery; CPCS = conventional phacoemulsification cataract surgery; M = male; F = female; FU = follow-up; NA = not available.
