# Supplementary material for: Efficacy and safety of femtosecond laser-assisted cataract surgery versus conventional phacoemulsification for cataract: a meta-analysis of randomized controlled trials
Source: Sci Rep. 2015 Aug 13;5:13123. doi: 10.1038/srep13123 (PMC4542520; doi:10.1038/srep13123)
Supplement: Supplementary Information [file srep13123-s1.doc]

**Supplementary information**

**Efficacy and safety of femtosecond laser-assisted cataract surgery versus conventional phacoemulsification for cataract: a meta-analysis**

Xiaoyun Chen^1,2^, Wei Xiao^1,2^, Shaobi Ye^1^, Weirong Chen^1,＊^, Yizhi Liu^1,＊^

1. State Key Laboratory of Ophthalmology, Zhongshan Ophthalmic Center, Sun Yat-sen University, Guangzhou 510060, People’s Republic of China;

2. These authors contributed equally to this work.

**^＊^Correspondence to:** Weirong Chen, M.D, Professor, Telephone: +86-020-87330293, Fax: +86-020-87333271, Email: [chenwrq@yahoo.com.cn](mailto:chenwrq@yahoo.com.cn); Yizhi Liu, M.D, Ph.D, Professor; State Key Laboratory of Ophthalmology, Zhongshan Ophthalmic Center, Sun Yat-Sen University, 54S Xianlie Road, Guangzhou 510060, People’s Republic of China; Telephone: +86-020-87330293; Fax: +86-020-87333271; Email: [yizhi_liu@aliyun.com](mailto:yizhi_liu@aliyun.com).

**Figure S1**

**Figure legend**

**Figure S1.**The risk of bias assessment of RCTs.

**Appendix 1**

**Literature-search strategy**

PubMed (up to Jun 15, 2015)

| #1 | femtosecond  Fields: All fields | 10232 |
| --- | --- | --- |
| #2 | bladeless  Fields: All fields | 45 |
| #3 | #1 OR #2  Fields: All fields | 10267 |
| #4 | phaco  Fields: All fields | 634 |
| #5 | phacoemulsification  Fields: All fields | 9619 |
| #6 | phakoemulsification  Fields: All fields | 9661 |
| #7 | #4 OR #5 OR #6  Fields: All fields | 9809 |
| #8 | cataract  Fields: All fields | 59580 |
| #9 | #3 AND #7 AND #8  Fields: All fields | 123 |

Embase (up to Jun 16, 2015)

| #1 | femtosecond  Fields: All fields | 5604 |
| --- | --- | --- |
| #2 | bladeless  Fields: All fields | 78 |
| #3 | #1 OR #2  Fields: All fields | 5669 |
| #4 | phaco  Fields: All fields | 898 |
| #5 | phacoemulsification  Fields: All fields | 11403 |
| #6 | phakoemulsification  Fields: All fields | 99 |
| #7 | #4 or #5 OR #6  Fields: All fields | 11645 |
| #8 | cataract  Fields: All fields | 85948 |
| #9 | #3 AND #7 AND #8  Fields: All fields | 162 |

**Cochrane Central Register of Controlled Trials (**CENTRAL, up to Jun 16, 2015)

| #1 | femtosecond  Fields: Search all text | 145 |
| --- | --- | --- |
| #2 | bladeless  Fields: Search all text | 9 |
| #3 | #1 OR #2  Fields: Search all text | 148 |
| #4 | phaco  Fields: Search all text | 206 |
| #5 | phacoemulsification  Fields: Search all text | 1859 |
| #6 | phakoemulsification  Fields: Search all text | 16 |
| #7 | #4 OR #5 OR #6  Fields: Search all text | 1898 |
| #8 | MeSH descriptor: [Cataract] explode all trees | 614 |
| #9 | #3 AND #7 AND #8  Fields: Search all text | 12 |

**Appendix 2**

**Table 1**. The elements of completely specified outcomes in the meta-analysis

| **Domain** | **Specific measurement** | **Specific metric** | **Method of aggregation** | **Time-point** |
| --- | --- | --- | --- | --- |
| Visual acuity | logMAR chart | value at a time-point | mean | post-op 1 W, 1-3 M, and 6 M |
| Corneal endothelial cell count | Specular microscopy | value at a time-point | mean | post-op 1W and 4-6 W |
| Central corneal thickness | AS-OCT | value at a time-point | mean | post-op 1D and 1 W |
| Mean phaco energy | Read from the machine | value at a time-point | mean | Intra-op |
| Mean phaco time | Read from the machine | value at a time-point | mean | Intra-op |
| Effective phaco time | Read from the machine | value at a time-point | mean | Intra-op |
| Circularity of capsulotomy | Ex situ capsulorhexis photography and manual evaluation by Photoshop | value at a time-point | mean | post-op 1 W |
| Complications (anterior capsule tear; macular edema; elevated IOP) | Surgeon reported; OCT;  Tenonometer | value at time-point | proportion | Intra-op; post-op 1 M; immediately and post-op 1 W |

AS-OCT= anterior segment optical coher­ence tomography; OCT=optical coher­ence tomography; IOP= intraocular pressure; W=week; M=month; D=day.
